# Application effect of computer-aided design combined with three-dimensional printing technology in autologous tooth transplantation: a retrospective cohort study

**DOI:** 10.1186/s12903-021-02030-z

**Published:** 2022-01-11

**Authors:** Shuang Han, Hui Wang, Jue Chen, Jihong Zhao, Haoyan Zhong

**Affiliations:** 1 The State Key Laboratory Breeding Base of Basic Science of Stomatology (Hubei-MOST) and Key Laboratory of Oral Biomedicine Ministry of Education, School and Hospital of Stomatology, Wuhan University, #237 Luoyu Road, Wuhan, 430079 China; 2 Department of Oral and Maxillofacial Surgery, School and Hospital of Stomatology, Wuhan University, Wuhan, China

**Keywords:** Tooth autotransplantation, Computer-aided design, 3D printing, Accuracy

## Abstract

**Background:**

The activity of donor periodontal membrane is the key factor of autologous tooth healing. The application of digital aided design, 3D printing model and guide plate in autotransplantation of tooth (ATT) is expected to reduce the damage of periodontal membrane and preserve the activity of periodontal membrane, so as to improve the success rate of ATT. This study tried to prove the role of digital technology in improving the success rate of ATT, although there are differences in model accuracy in practice.

**Methods:**

We included 41 tooth autotransplantation cases which assisted by 3D-printed donor models and surgical guides and divided them into two groups in accordance with whether the donor tooth could be placed successfully after the preparation of alveolar socket guided by the model tooth. Then, we compared and analyzed the preparation time of alveolar socket, extra-alveolar time, and number of positioning trials of the donor tooth between the two groups. We also included a comparison of the in vitro time of the donor tooth with that of 15 min. The incidence of complications was included in the prognostic evaluation.

**Results:**

The mean preparation time of the alveolar socket, mean extra-alveolar time of donor tooth, and mean number of positioning trials with donor tooth of 41 cases were 12.73 ± 6.18 min, 5.56 ± 3.11 min, and 2.61 ± 1.00, respectively. The group wherein the donor tooth cannot be placed successfully (15.57 ± 6.14 min, 7.29 ± 2.57 min) spent more preparation time of alveolar socket and extra-alveolar time than the group wherein the donor tooth can be placed successfully (9.75 ± 4.73 min, 3.75 ± 2.57 min). The number of positioning trials with the donor tooth of the group wherein the donor tooth cannot be placed successfully (3.19 ± 0.75) was higher than that of the other group (2.00 ± 0.86). There was no significant difference in survival rates between the two groups.

**Conclusions:**

Compared with the traditional tooth autotransplantation, the introduction of computer-aided design combined with 3D printing of the model tooth and surgical guides evidently shortens the preparation time of the alveolar socket and the extra-alveolar time of the donor tooth and reduces the number of positioning trials with the donor tooth regardless of the shape deviation between the model and actual teeth.

## Background

The aim of the autotransplantation of tooth (ATT) is to replace a lost tooth with a functional tooth within the same patient for the restoration of the masticatory and aesthetic functions of the recipient site. ATT can be used to treat dental defects caused by missing teeth, deep caries, poor endodontic prognosis, and periodontitis [1]. The success rate of the contemporary ATT was close to that of implant [2], with about 90% in patients younger than 30 years old and 80% in patients older than 30 [3]. In terms of economic cost, self-adaptation, and physiological feeling, ATT has absolute advantages over artificial implants and many other kinds of restorative procedure [1, 4].

The traditional ATT uses the donor tooth to prepare alveolar socket directly in the process of operation, which increases the extra-alveolar time of donor tooth and inevitably causes mechanical damage to the periodontal membrane on the root of the donor tooth, thus reducing the vitality of periodontal membrane cells. The most critical factor for the success of ATT is the presence of viable periodontal ligament (PDL) on the surface of donor tooth root [1].

Lee et al. [5] used spiral CT and computer-aided rapid prototyping (CARP) to guide surgery by producing a life-sized resin jaw model and an actual-sized tooth model to solve these problems. Keightley et al. [6] used CBCT and CARP for the first time to guide a case of ATT. In the same year, Shahbazian et al. used the 3-matic program to make a 3D model and stereolithographic model to guide the ATT [7]. These clinical trials showed that the extra-alveolar time of the donor tooth is less than 15 min and that the probability of root resorption is remarkably reduced when the donor tooth is isolated for less than 15 min [8]. These experiments showed that the application of computer-aided design (CAD) and 3D printing technology in the ATT can achieve good expectations [6]. In recent years, the 3D models of donor tooth are widely introduced in ATT [9].

However, in the actual operation process, the surgeon who only uses the 3D model tooth to assist in preparing the alveolar socket cannot confirm the position of the donor tooth designed by the computer before. Thus, the position and occlusion of the 3D model tooth can hardly be consistent with the original design. Moreover, we found that some of the model teeth printed with the volume ratio of 1:1 to the donor teeth simulated by the computer are different from the actual donor teeth. Thus, parts of the donor teeth cannot be placed successfully. Thus, a secondary preparation is needed, which may prolong the operative time and affect the prognosis of autotransplantation.

All included cases use the CAD to simulate the implant site, achieve relatively appropriate occlusal of the donor tooth, and make 1:1-sized 3D models of the donor teeth and surgical guide plates for the accurate control of direction and depth of the preparation of the alveolar socket. After 1 year follow-up, the clinical effect of this technique is observed, and the preparation time of alveolar socket, extra-alveolar time, and number of positioning trials of the donor tooth are analyzed to provide reference and assistance for the application of digital technology in ATT.

## Methods

A total of 41 consecutive cases of ATT completed in the Department of Oral Surgery of the Hospital of Stomatology Wuhan University from May 2019 to January 2020 were included. 3D model and surgical guide plates were preformed using a CAD on the basis of preoperative CBCT data in all cases to guide the preparation of alveolar socket during surgery.

**Fig. 1:**
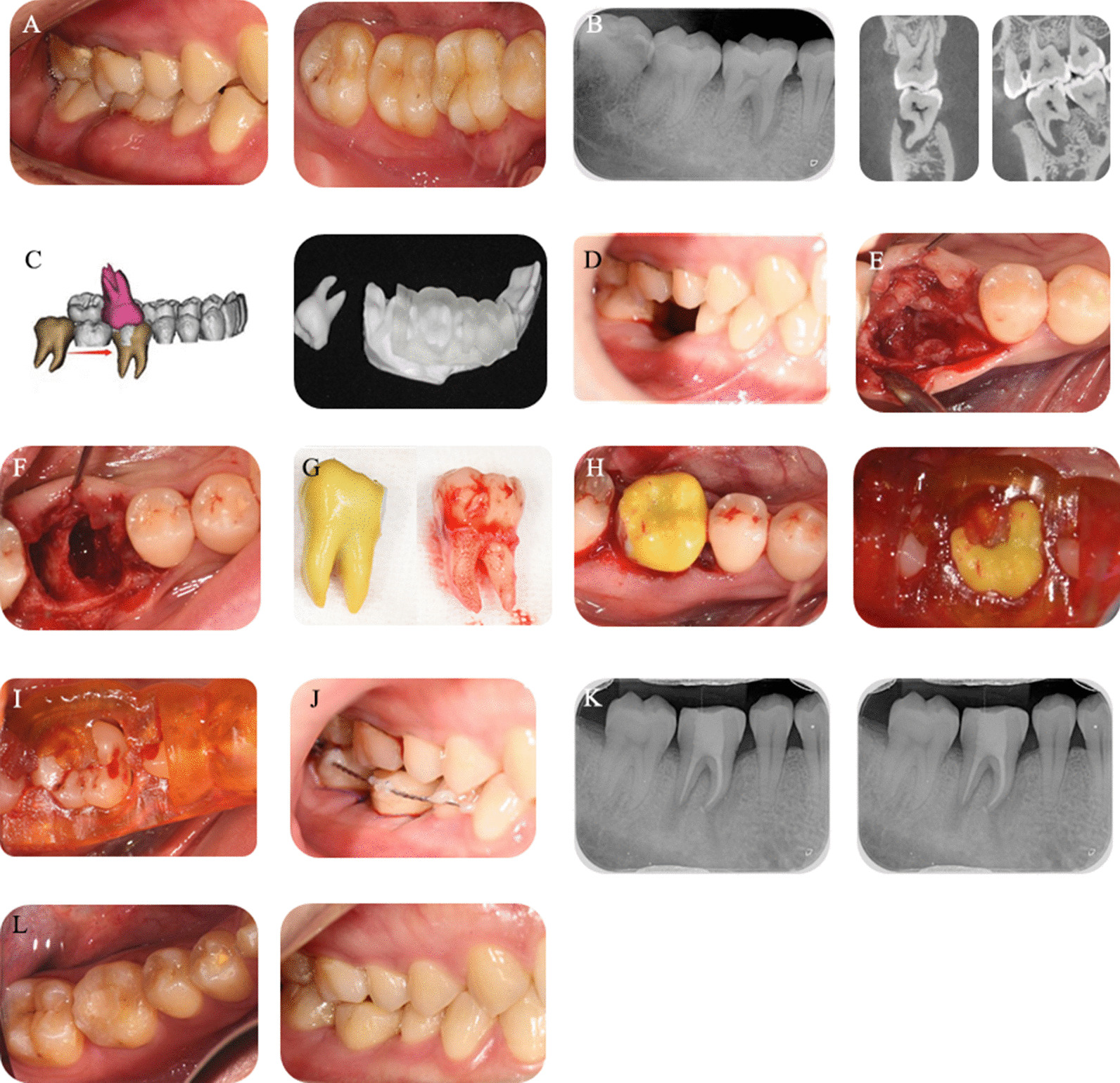
**a** Buccal and occlusal initial situations of the recipient site. **b** Initial X-ray and CBCT showing bone defect around the mesial root of the right mandible first molar. **c** Three-dimensional reconstruction and simulation implantation of third molar of right mandible performed on the software and printed replica of the third molar of right mandible and the guiding template. **d** One month after 46 removal. **e** Incision of the recipient site. **f** Preparation of the alveolar socket with the help of the model tooth and guiding template. **g** Model and donor teeth. **h** Placement of the replica and replica with the guidance of template. **i** Placement of the donor tooth. **j** Donor and adjacent teeth fixed using steel wire and adhesive. **k** 1- and 3-month review of the recipient site. **l** Buccal and occlusal situation of the donor tooth and recipient site

Before surgery, CBCT was taken by the same CT machine (Newtom VGI, Quantitative Radiology, Verona, Italy) in all included cases, and parameters were consistent (Fig. 1b). The image output format was DICOM 3.0, and the resolution of the CT machine was 0.30 mm. The image analysis and processing software were NewTomNNT. Donor models and surgical guides were designed by one doctor through the Mimics Medical 20.0 (Materialise, Leuven Belgium)/Materialise 3-matic 11.0 (Materialise, Leuven Belgium) and printed by the Formlabs Form2 SLA photocurable 3D printer (Formlabs, Massachusetts America) by using Photopolymer Resin White FLGPWH03 (Formlabs, Massachusetts America)/Photopolymer Resin Clear FLGPCL02 (Formlabs, Massachusetts America). (Fig. 1c). The printer thickness was 0.05 mm. The photocuring time of the resin was 15 min. The model was disinfected preoperatively with 0.5% povidone iodine solution.

Surgeries were performed by one experienced doctor, and procedures were consistent. The model tooth was assisted in preparing the alveolar socket until it was in place, and the surgical guide plate was used to confirm that the model tooth was positioned in the location designed before the surgery (Fig. 1f, h). The donor tooth was completely extracted, and the root was wrapped around with concentrated growth factors, which were isolated from the patient’s own venous blood before operation and implanted into the well-prepared alveolar socket. The guide plate helped guide the direction of the alveolar socket preparation and confirmed that the donor tooth was in place (Fig. 1i). In the presence of deviation between the actual donor tooth and the model tooth, the donor tooth could not be placed successfully, and further preparation of the alveolar socket was needed. The donor tooth should be temporarily stored in normal saline solution at 4 °C.

The implanted tooth was fixed with elastic wire and fluid resin (Fig. 1j). The occlusion of the proximal and distal adjacent teeth was elevated with glass ionomer cement. The root canal therapy (RCT) was performed 2–4 weeks after operation for donor tooth with closed apical foramen(Fig. 1k). The retainer wire was removed after the completion of RCT. Regular return visits were conducted.

The following information was extracted: gender, age, position of donor tooth, diagnosis of the recipient site, root development, preparation time of alveolar socket, extra-alveolar time of donor tooth, number of positioning trials with the donor tooth, successful placement of the donor tooth, performance of RCT after surgery, and imaging data.

Data were analyzed using the IBM SPSS 26.0 and represented in the “${\bar{x}}$ ± *s*” form. The single-sample t-test was carried out on the extra-alveolar time data, and the test value was 15. The independent sample Mann–Whitney U test was used to compare the preparation time of alveolar socket in the recipient site, extra-alveolar time, and number of positioning trials with the donor tooth between the groups wherein the donor tooth can and cannot be placed successfully. The test level was *α* = 0.05 on both sides. And STROBE guidelines was adopted in our study.

## Results

### Situation analysis of the donor tooth

**Fig. 2:**
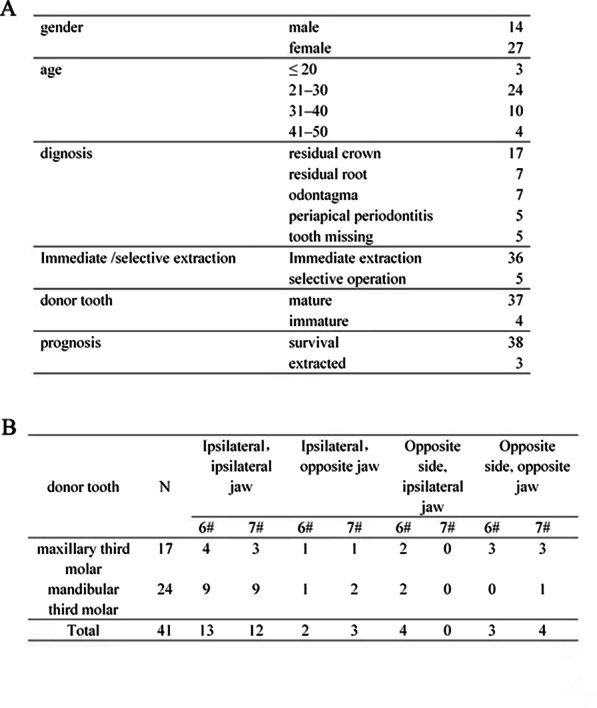
**a** The clinical features of 41 cases. **b** Conditions of donor tooth and recipient sites

In this study, 14 males and 27 females were included. The mean age was 28.68 ± 6.75 years. The oldest age was 48 years, and the youngest age was 18 years. The clinical features are shown in Fig. 2. All donor teeth were third molars. The RCT was completed in 29 cases 2–4 weeks after transplantation, whereas six cases underwent root tip resection and iRoot BP backfill because of root fracture during the extraction. In four cases of immature donor tooth, one case was fully developed, and the pulp healed. Three other cases completed RCT in the following 2–3 months due to apical periodontitis. Periodontal healing was observed in 38 cases half a year after the surgery. Periapical periodontitis occurred in one case due to delayed RCT, and external root resorption occurred in one case because of incomplete RCT. One case had a tooth extracted due to infection without timely RCT. There was no significant difference in the retention rate between the two groups.

### Analysis of the preparation time of alveolar socket, extra-alveolar time, and number of positioning trials of the donor tooth

**Fig. 3:**
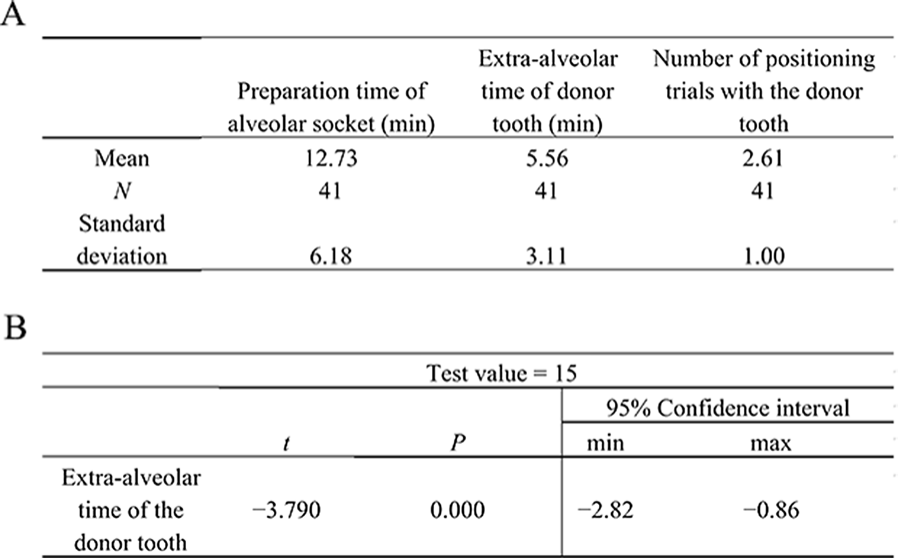
Analysis of the preparation time of alveolar socket, extra-alveolar time, and number of positioning trials of the donor tooth. **a** Analysis of the preparation time of alveolar socket, extra-alveolar time of donor tooth, and number of positioning trials with the donor tooth. **b** One-sample test of the extra-alveolar time of the donor tooth

The mean preparation time of the alveolar socket of 41 cases was 12.73 ± 6.18 min, and the shortest and longest preparation times were 3 and 30 min, respectively. The mean extra-alveolar time of donor tooth was 5.56 ± 3.11 min, and the shortest and longest extra-alveolar times were 1 and 15 min, respectively. The mean number of positioning trials with the donor tooth was 2.61 ± 1.00, and the minimum and maximum numbers were 1 and 5, respectively (Fig. 3a).

The data of the extra-alveolar time of the donor tooth conformed to the normal distribution. The single-sample t-test was conducted on the data, and the test value was 15 (Fig. 3b). The difference between the extra-alveolar time of the donor tooth and 15 min was statistically significant (*P* < 0.05).

### Comparison of the data between groups wherein the donor tooth can and cannot be placed successfully after the alveolar socket was prepared with the help of 3D model tooth and guide plate

**Fig. 4:**
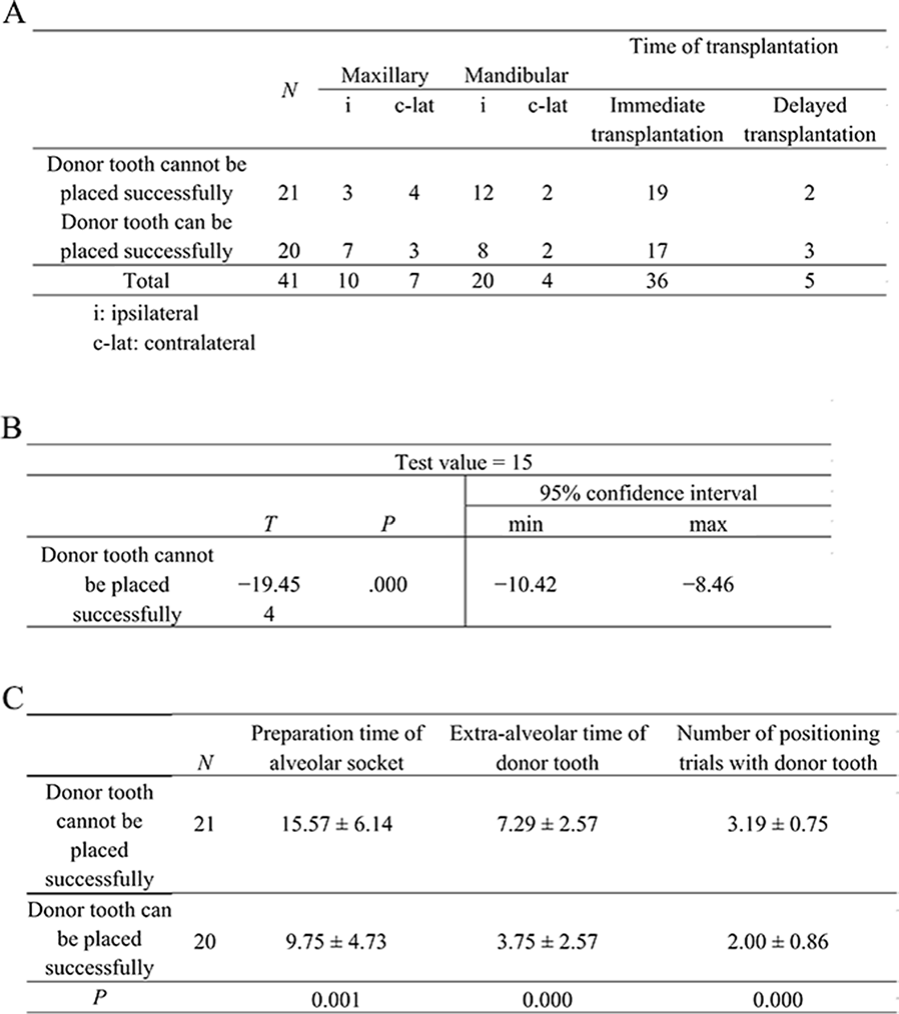
Information of groups wherein the donor tooth can and cannot be placed successfully after the alveolar socket was prepared with the help of 3D model tooth and guide plate. **a** Analysis of the donor tooth in position after preparation of alveolar socket with the assistance of 3D model tooth. **b** Single-sample test of the extra-alveolar time of donor tooth in the group wherein the donor tooth cannot be placed successfully. **c** Data comparison of preparation time of alveolar socket, extra-alveolar time of donor tooth, and number of positioning trials with the donor tooth between two groups

In 21 cases (51.2% of the total cases), the donor tooth cannot be placed successfully after the alveolar socket was prepared with the assistance of 3D model (Fig. 4a).

In the group wherein the donor tooth cannot be placed successfully, the mean preparation time of the alveolar socket, mean extra-alveolar time of donor tooth, and mean number of positioning trials with the donor tooth were 15.57 ± 6.14 min, 7.29 ± 2.57 min, and 3.19 ± 0.75, respectively. The data of the donor tooth extra-alveolar time in the group wherein the donor tooth cannot be placed successfully conformed to normal distribution. The single-sample t-test was conducted on the data, and the test value was 15 (Fig. 4b). A significant difference was present between the extra-alveolar time of donor tooth in the group wherein the donor tooth cannot be placed successfully and 15 min (*P* < 0.05).

The mean preparation time of the alveolar socket, mean extra-alveolar time of donor tooth, and mean number of positioning trials with the donor tooth in the group wherein the donor tooth can be placed successfully were 9.75 ± 4.73 min, 3.75 ± 2.57 min, and 2.00 ± 0.86, respectively (Fig. 4c).

**Fig. 5:**
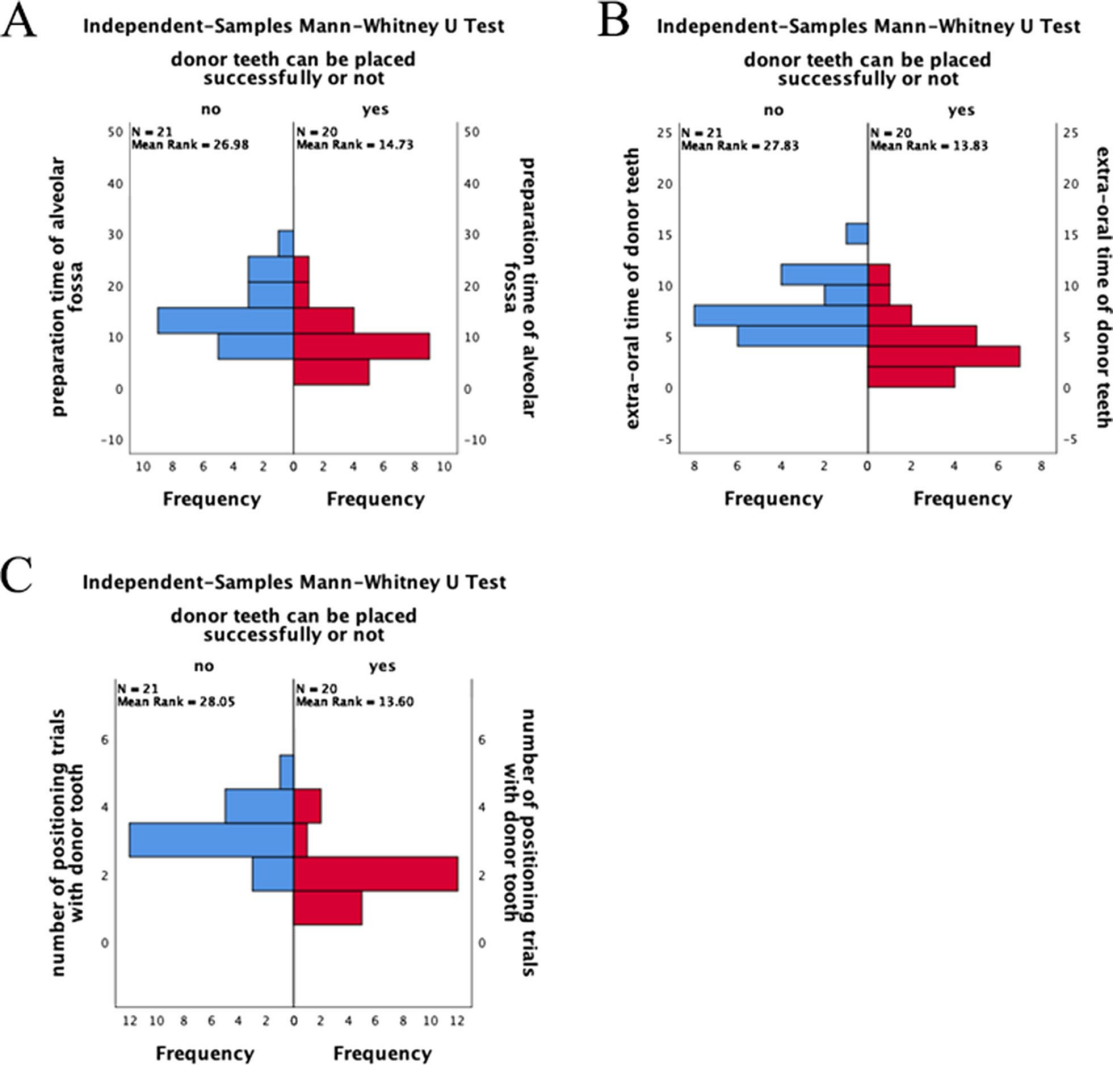
Comparison of the data between groups wherein the donor tooth can and cannot be placed successfully after the alveolar socket was prepared with the help of 3D model tooth and guide plate. **a** Independent sample Mann–Whitney U test of the preparation time of the alveolar socket between two groups. **b** Independent sample Mann–Whitney U test of the extra-alveolar time of the donor tooth between two groups. **c** Independent sample Mann–Whitney U test of the number of positioning trials with the donor tooth between two groups

The preparation time of the alveolar socket, extra-alveolar time of donor tooth, and number of positioning trials with the donor tooth of the group wherein the donor tooth can be placed successfully were statistically different with the group wherein the donor tooth cannot be placed successfully (*P* < 0.05) (Fig. 5).

## Discussion

ATT, the best restorative way of biocompatibility, has absolute advantages over dental implants in terms of the formation of periodontal membrane healing and even pulp healing [10, 11]. Their aesthetics and usability are also superior to other restorative methods [12]. However, the operation process of ATT is difficult and complicated, therefore, many dentists are willing to choose a simple method, such as dental implant or fixed partial denture [1, 13].

An important factor for the success of ATT is to maximize the integrity and vitality of the periodontal membrane on the root surface of the transplanted teeth [10]. A short extra-alveolar time of the donor tooth decreases the likelihood of damage to periodontal membrane cells on the root surface and increases the likelihood of forming a normal periodontal membrane after transplantation [6, 14]. Thus, the transplanted teeth can perform normal occlusal and masticatory functions [11, 15]. With increased extra-alveolar time of donor tooth, the vitality of periodontal membrane cells decreases, and the proportion of postoperative cemental healing increases, resulting in decreased success rate [14]. At present, no uniform standard exists for the safe time of the extra-alveolar time of the donor tooth. Hammarström et al. used two different extra-alveolar times for transplanted teeth. After initial ankylosis, the ankylotic area does not increase in a 15 min extra-alveolar period group, whereas progressive ankylosis is observed in the 60-min extra-alveolar period group [8]. Andreasen and his colleagues observed normal PDL healing in more than 80% of cases after an extra-alveolar time of 18 min [16]. In our retrospective study, 15 min was set as the test value of the independent t-test.

Another important factor affecting the prognosis of ATT is the proper distance between the alveolar socket and the donor tooth [15] which can improve the blood and nutrition supply of periodontal membrane cells and avoid the physical extrusion to the periodontal membrane for improved success rate of ATT [5, 17]. Therefore, the preparation of the recipient site accurately and reduction in the number of positioning trials with the donor tooth are also our goals [16].

A digital technology assisted ATT treatment approach has been developed, including CBCT analysis, simulation, and preparation of 3D model and guide plate, to reduce the extra-alveolar time of donor tooth, prevent potential damage to the PDL, and prepare the alveolar socket accurately [1]. We used CBCT to analyze the morphology of the donor tooth and recipient site to simulate the transplantation process on the Mimics software, which can assess the feasibility of the surgery intuitively and estimate the possibility of invasion of anatomical structures, such as the alveolar neural tube or the maxillary sinus cavity [5]. It helps increase the predictability of treatment outcomes and reduces the difficulty of doctor–patient communication [18]. The preoperative 3D donor tooth model of 1:1 size is designed and printed [15]. Thus, in the process of preparing alveolar socket, the 3D model tooth is used to replace the donor tooth for trial implantation, shorten the extra-alveolar time, reduce the number of positioning trials with the donor tooth, and avoid the periodontal membrane injury [7, 12, 19–21]. The 3D model can improve the efficiency in preparing the alveolar socket and prevent excessive preparation. The digital guide plate can be consistent with the occlusal surface of the 3D model to guide the direction and depth of the preparation of the alveolar socket to achieve the accurate preparation of the alveolar socket and ensure that the position after the preparation is exactly the position we designed before operation. Results showed that the CAD combined with 3D model teeth and guide plate was helpful in shortening the preparation time of alveolar socket and extra-alveolar time of donor tooth and reducing the number of positioning trials with the donor tooth. The mean extraction time of donor tooth (5.56 ± 3.11) was far less than 15 min (*P* < 0.05). A previous study showed that the probability of root resorption is remarkably reduced when the donor tooth is isolated for less than 15 min [8].

The application of 3D printing technology in ATT has achieved good results, but this technology still has some limitations. For example, it takes about 1.5–2 h in each case at the segmentation, simulation and production stage. A shorter time is expected by improving the accuracy of automatic identification of the software and increasing the printing speed. The model teeth of some cases are not completely consistent with the shape of the donor tooth [5, 20]. We found the same problem during the surgery. In this study, 21 cases with some differences in the shape of the 3D model with the actual donor teeth were included. Thus, the second preparation of the alveolar socket was needed, which led to increased preparation time of the alveolar socket, extra-alveolar time of donor tooth, and number of positioning trials with the donor tooth. Compared with 20 cases, which could successfully place the donor tooth after preparation, cases which could not successfully place spent more preparation time of alveolar socket, extra-alveolar time of the donor tooth and had more positioning trials with the donor tooth (*P* < 0.05). The mean extra-alveolar time of the donor tooth in the group wherein the donor tooth cannot be placed successfully (7.29 ± 2.57) was still less than 15 min (*P* < 0.05). And even if there were differences in the models, the survival rate of this group was not affected, which meaned that the model differences only extended the time of donor tooth removal and alveolar socket preparation within a certain range, but would not reduce the survival rate of tooth.

From the acquisition of CT data to the implementation of the surgery, a number of potential sources of error is present at each stage of the process [17]. We speculated that the following factors might explain the difference between 3D models and donor tooth.

First, the accuracy of CT may affect the accuracy of 3D modeling. The CT scanning layer thickness and voxel affect the image resolution [22]. A high resolution results in high accuracy of observation, measurement, and outline of the structure. The gray value of the image affects the doctor’s judgment of the tissue structure. The position of the patient’s jaw, whether the mouth is kept in the correct opening position, and whether the jaw is moved will affect the final accuracy of the CT [17, 23]. Second, when the Mimics software is used to simulate the tooth transplantation operation, the shape of the donor tooth should be manually outlined, separated, and reconstructed [5, 15], which may lead to errors [24]. After 3D reconstruction, the root of the donor tooth model is rough and needs to be smoothed [17], which may result in a deviation to the root morphology of the model tooth. Although model teeth are made 1:1 with the donor tooth, the periodontal membrane has a certain thickness, which may result in the unsuccessful placement of the donor tooth. Third, the oxidation polycondensation is a common problem of photocurable resin materials [25]. A certain time interval is observed between the production of 3D model teeth and the use of 3D model teeth. The aggravation of the deformation of resin is also a problem that should be considered.

In addition, the computer-aided rapid prototyping technology produces complex 3D physical models by selective modification. Print layer thickness is one of the key parameters, It will affect the accuracy of the replica [26, 27].

In addition, the horizontal layer at a time with stepwise submergence along the vertical axis, during this process, the precision of the printer may also lead to the deviation of the model [17].

## Conclusions

The application of digital guide plate and donor model in ATT can significantly shorten the preparation time of the alveolar socket in the recipient site and extra-alveolar time of donor tooth and will reduce the number of positioning trials with the donor tooth. Even if the current technology has certain limitations in accuracy, it still helps clinicians to improve or ensure the retention rate of ATT.

## Data Availability

The datasets used and/or analysed during the current study are available from the corresponding author on reasonable request.

## Abbreviations

CBCT: Cone beam computed tomography
PDL: Periodontal ligament
